# Eating Behavior and Factors of Metabolic Health in Primary Schoolchildren: A Cross-Sectional Study in Greek Children

**DOI:** 10.3390/nu15163592

**Published:** 2023-08-16

**Authors:** Aristea Gioxari, Charalampia Amerikanou, Sevasti Peraki, Andriana C. Kaliora, Maria Skouroliakou

**Affiliations:** 1 Department of Nutritional Science and Dietetics, School of Health Science, University of the Peloponnese, Antikalamos, 24100 Kalamata-Messinia, Greece; a.gioxari@uop.gr (A.G.); nds19117@go.uop.gr (S.P.); 2 Department of Dietetics and Nutritional Science, School of Health Science and Education, Harokopio University, 70 El. Venizelou Ave., 17671 Athens, Greece; amerikanou@windowslive.com (C.A.); mskour@hua.gr (M.S.)

**Keywords:** primary school children, breakfast at home, metabolic health, cardiorespiratory fitness, handgrip strength, antioxidant status

## Abstract

Childhood obesity has been associated with altered blood lipids and bad eating habits. In this cross-sectional study, we assessed cardiorespiratory fitness and metabolic health markers in regard to weight status and dietary habits in schoolchildren. In 134 children (6–11 years), we conducted: (1) Anthropometry, namely z-score BMI (z-BMI), waist-to-height ratio (WHtR), and body composition analysis. (2) Measurements of handgrip strength (HGS), resting metabolic rate (RMR) and VO_2_max. (3) Quantification of blood lipids and antioxidant vitamins A, E, C. (4) Eating breakfast assessment. About 35% of children were overweight/obese. The z-BMI positively correlated with WHtR (r = 0.637, *p* < 0.001), and adversely correlated with fat-free mass (r = −0.728, *p* < 0.001) and vitamin E (r = −0.286, *p* < 0.001). RMR and VO_2_max were greater in normal weight children compared to those with overweight/obesity (*p* < 0.001). HGS did not differ between these groups, but was negatively correlated with dyslipidemia as shown by TG/HDL-C ratio (r = −0.224, *p* = 0.037). According to regression analysis, eating breakfast routinely at home was positively associated with RMR and adversely associated with z-BMI. Hence, regular breakfast consumption at home may improve RMR in kids. Cardiorespiratory fitness and physical strength are key modulators of metabolic health in Greek children added to a social determinant of health i.e., eating breakfast at home.

## 1. Introduction

Childhood obesity has emerged as a global public health concern with its prevalence rising drastically both in developed and developing countries. According to the World Health Organization (WHO), more than 340 million children and adolescents aged 5–19 were overweight or obese in 2016 [1]. It is well confirmed that obesity in adults is highly associated with reduced antioxidant status, as indicated by inadequate circulating antioxidant vitamins i.e., carotenoids (vitamin A), tocopherols (vitamin E) and ascorbic acid (vitamin C) [2]. Recently, this redox state imbalance has been also observed in children and adolescents with overweight/obesity, accompanied by an altered blood lipid profile such as reduced levels of high-density lipoprotein-cholesterol (HDL-C) and high triglyceride (TG) concentration [3]. There is evidence that adiposity during childhood is characterized by subclinical inflammation with elevated secretion of proinflammatory adipokines contributing to increased oxidative stress [4,5]. In turn, systemic inflammation and oxidative stress during childhood are associated with the development of obesity-related metabolic complications such as cardiovascular diseases (CVD) in adult life [5,6]. According to a recent case-control study in 1444 children, metabolically unhealthy participants demonstrated lower ratios of vitamin A/TG and vitamin E/TG in plasma compared to their metabolically healthy counterparts, indicating the pivotal role of these antioxidant vitamins in metabolic health [7].

Low cardiorespiratory fitness (CRF) is directly linked to excess adiposity and is a major risk factor for cardiovascular disease in adults [8]. CRF is assessed by measuring maximal oxygen consumption (VO_2_max), which is the maximum capacity of the body to transport and use oxygen during physical activity [8]. Improvement in CRF of adults has been associated with ameliorated body composition i.e., visceral fat and total fat mass, and lower waist and hip circumferences [8,9,10], but for children, data are limited. Very recently, it has been suggested that CRF is a potent indicator of cardiovascular health in children and adolescents as well [11]. Based on epidemiological data, only 40% of 12–15-year-olds in the US have a healthy CRF [12]. What is more, CRF has been currently associated with handgrip strength (HGS), which in turn affects all-cause mortality and CVD risk in young and older adults [13,14]. There is now evidence that low HGS may be associated with poor cardiometabolic outcomes in children and adolescents too [15]. In a recent observational study in 2256 children and adolescents aged 5–18 years, HGS-to-body mass index (BMI) presented a positive correlation with cardiorespiratory fitness [16].

Eating habits may influence metabolic health. A higher meal frequency seems to be predictive for a lower weight status in children, as indicated by z-scores of body mass index (z-BMI) for age and waist circumference to height ratio (WHtR) [17]. In addition, skipping breakfast has been associated with a 43% higher risk of developing obesity [18,19]. It is not doubtful that eating habits are formed during childhood and are crucial for a child’s development [20], while the home food environment plays a fundamental role in attaining a healthy body weight [21].

Nevertheless, data regarding the role of CRF and HGS in overweight/obesity and how these factors correlate to adiposity, antioxidant status, blood lipid profile and eating habits in children in Greece are limited. Therefore, in the present cross-sectional study we aimed to investigate differences between normal weight children and children with overweight/obesity, regarding fitness indicators (CRF, HGS), biochemical markers (antioxidant vitamins, lipid profile), and dietary habits such as breakfast consumption at home or snacking at school.

## 2. Methods and Materials

### 2.1. Ethics Approval

The research protocol was submitted to the Scientific and Ethical Committee of “IASO Maternity Hospital, Obstetrics—Gynecology (Attica, Greece)” and was authorized with an approval code #J-31052019. Throughout the study, the principles of Helsinki Declaration 1964 were adhered, and the General Data protection Regulation (EU) 2016/679 was assured. To this end, a written informed consent was delivered to all parents (or legal guardians) of recruited children, and a copy of the signed consent was handed to each parent.

### 2.2. Study Design

The present work is a cross-sectional study, in which community children from Attica (Greece) were enrolled during June and July 2019. Parents or legal guardians were invited to take part in the study through written announcements (posters), electronic invitations and social media posts by the collaborating primary schools. Those who agreed to participate were provided with a detailed information leaflet describing the aims, methods, benefits, and potential hazards of the study.

Participants’ inclusion criteria were set as follows:

(a) community children, aged 6 to 11 years, living in Attica (Greece);
(b) apparently healthy children without seasonal infection, chronic or life threatening diseases (as described below);
(c) parental consent.

Exclusion criteria were:

(a) children with infections (e.g., seasonal influenza), chronic diseases (e.g., celiac disease, inflammatory bowel diseases, kidney disease, malabsorption, parathyroid diseases), pediatric metabolism disorders (e.g., type 1 diabetes) or life threating diseases (e.g., cancer, congenital disorders);
(b) children with cognitive disorders (e.g., autism, attention-deficit/hyperactivity disorder);
(c) children under medication treatment that could affect the outcomes of the study;
(d) children following a vegetarian or a vegan diet;
(e) children receiving nutritional supplements including vitamins and minerals;
(f) parental refusal to consent.

### 2.3. Clinical, Laboratory and Nutritional Data

All measurements and data collection were conducted at IASO Maternity Hospital (Attica, Greece) under the supervision of the appointed physician and the collaborating researchers. Parents (or guardians) visited along with their child hospital’s facilities in three separate days within two weeks for: (1) medical history record, anthropometric measurements and blood analysis; (2) CRF and HGS assessments; and (3) eating behavior evaluation, respectively. Prior to visiting the clinical site, parents (or guardians) were instructed to ensure that their child was healthy, fully rested, joyful and well hydrated. Throughout each test parents (or guardians) were present, while children were advised to express themselves if they would feel discomfort during measurements. All children were verbally encouraged by the researchers to do their best during the tests providing positive feedback.

#### 2.3.1. Day 1: Anthropometry and Blood Analysis

Medical history: The appointed pediatrician recorded children’s medical history from parents (or guardians) including general information, as well as disease specific data (i.e., allergies, medical condition, drug treatment, nutrient supplementation).

Anthropometry: Experienced dieticians performed measurements of body weight (BW), height (Ht), waist circumference (WC), as well as body composition and resting metabolic rate (RMR).

More specifically, Ht was measured on a standard stadiometer without shoes to the nearest millimeter (Seca Mode 220, Hamburg, Germany), and BW on a flat scale with light clothing to the nearest 0.1 kg. BMI was calculated as the ratio of BW (kg) to the square of Ht (m^2^). To assess growth and weight status, z-scores of Ht-for-age, BW-for-age and BMI-for-age were calculated according to WHO growth reference data for boys and girls aged 5 to 19 years [22]. WC was measured with a non-stretch but flexible tape on minimal clothing. For defining abdominal adiposity, the WHtR was used with a cut-off ≥0.50 [23].

Body composition i.e., fat mass (FM), fat-free mass (FFM) and total body water (TBW), was analyzed with air displacement plethysmography (Bodpod^®^ Body Composition Tracking Systems, Life Measurement, Inc., Rome, Italy) following the manufacturer’s instructions. Measurement was performed on minimal clothing. Preparation for the Bodpod test included overnight fasting and abstaining from rigorous activities at the day before the examination [24]. Body composition indicators, FM, FFM and TBW were expressed as kg or % BW.

An open-circuit indirect calorimetry of computerized metabolic system with ventilated canopy (Quark RMR, Cosmed, Rome, Italy) was applied to measure RMR (kcal/day) based on measurements of gas exchange, namely oxygen consumption (VO_2_) and carbon dioxide production (CO_2_) [25]. The oxygen sensor was calibrated before each measurement with the use of mixed reference gases of known composition. Measurements were conducted after overnight fasting and prior to the measurement children were asked to rest in the recumbent position for 10 min in a thermoneutral environment. Then, the canopy was placed over their head, and they were asked to breathe normally for 25 min avoiding hyperventilation or falling asleep during the test. RMR output was also expressed in relation to BW, namely, kcal/kg of BW/day.

Blood analysis: Blood withdrawal (8 mL) from each child was performed after overnight fasting. To isolate serum, whole blood was allowed to clot at room temperature for 20 min followed by centrifugation at 3000 rpm for 10 min (4 °C). For plasma isolation, whole blood was collected in ethylenediaminetetraacetic acid (EDTA)-filled tubes prior to centrifugation.

Total cholesterol (TC), low-density lipoprotein-cholesterol (LDL-C), HDL-C and TG were quantified in sera with an automatic biochemical analyzer using manufacturer’s reagents (Cobas 8000 modular analyzer, Roche Diagnostics GmbH, Mannheim, Germany). A reversed-phase high performance liquid chromatography (HPLC) system (model 1050, Agilent Technologies, Waldbronn, Germany) coupled with ultraviolet (UV) and fluorescence (FL) detectors, quaternary pump, auto-sampler and data analysis software, was used to quantify antioxidant vitamins A (retinol), E (alpha-tocopherol) and C (ascorbic acid) in plasma samples, according to previously published methods [26,27]. All blood analyses were performed in freshly drawn blood samples.

#### 2.3.2. Day 2: CRF and HGS

CRF: Τhe appointed pediatrician was responsible for conducting the maximal oxygen consumption (VO_2_max) examination test. Measurement of VO_2_max was performed on a treadmill (Woodway ELG 55, Waukesha, Germany) according to a modified Balke protocol for children [28]. Initially, children were asked to warm up on the treadmill at a self-selected speed for 10 min. Next, children performed an exercise test to fatigue at a constant speed of 5.0 mph consisting of 2 min stages of progressively increasing the incline of the treadmill by 2.5%. VO_2_max was determined as the highest oxygen consumption attained during the test, upon fulfillment of three out of four criteria: (a) VO_2_ reached a plateau; (b) respiratory exchange ratio (RER) was greater than 1.2; (c) rating of perceived exertion (RPE) was greater than 17; heart rate (HR) approached the maximum age-predicted heart rate (±10 beats/min) [29]. Output of VO_2_max was expressed as mL O_2_ per kg of BW per min (mL O_2_/kg/min).

HGS: The appointed dieticians conducted two HGS measurements of the dominant hand, each on a separate day. The average of both measurements was calculated and HGS output was expressed as kg. To this scope, the Jamar Plus+ Digital Hand Dynamometer (Patterson Medical, Warrenville, IL, USA) was used. Before HGS test, children were asked to familiarize themselves with the dynamometer by grasping the handle, adjusting grip to the handle and performing 2–3 tests. Then, measurements were preformed according to the standard procedures recommended by the American Society of Hand Therapists (ASHT) [30]. More specifically, children were asked to sit upright on a chair with their feet supported on the ground and to place their tested hand on the table in front, maintaining the arm position during the test. Shoulders had to be slightly abducted and neutrally rotated, the elbow in 90° of flexion, the forearm in 0° between pronation and supination, and the wrist in neutral resting position [31,32]. Children were then asked to squeeze the grip continuously for 3 sec. Children were not aware of the HGS test outcome as the display of the dynamometer always faced the researcher. The second HGS measurement was performed on the third visit, before nutritional data collection.

#### 2.3.3. Day 3: Nutritional Behavior Assessment

Children’s behavior regarding breakfast and snacking at school during the last month was assessed by applying the KIDMED questionnaire via face-to-face interviews with the parents (or guardians) and children [33]. Questions denoting a negative connotation with respect to the Mediterranean diet were assigned a value of −1 and those with a positive aspect +1 [33].

All children invited to participate were attending public schools that have food canteens. Children who positively responded of eating breakfast daily (or most of the days) were further asked if they consumed breakfast at home or not. Children who consumed breakfast on a regular basis but apart from home were also recorded.

## 3. Statistical Analysis

Statistical analysis was performed using the SPSS software for Windows (Version 26, Armork, NY, USA, IBM Corporation). Significance level was set at *p*-value ≤ 0.05. The Kolmogorov–Smirnov test was used to assess normality of variable distribution. Dichotomous variables were presented as counts (N) or relative frequencies (N %) and quantitative variables as mean and standard deviation of the mean (SD).

Children were categorized according to z-BMI, as underweight (z-BMI < −2), normal weight (z-BMI −2 to 0.99) or overweight/obese (z-BMI ≥ 1) [34]. Only two children were scored as underweight, and were excluded from further statistical analysis. The independent-samples *t*-test was conducted to compare variable means between the normal weight and the overweight/obesity group. To compare eating behavior frequencies between groups, the chi-square test was used.

Children were also categorized according to the frequency of having breakfast. All children who reported eating breakfast daily (or most of the days) consumed this meal exclusively at home. Conversely, those reporting not having breakfast at home, all skipped this meal in the morning. Therefore, two categories of breakfast frequency were used: “daily/most of the days” or “sometimes/no”. The independent-samples *t*-test was performed for the comparison of means between the two groups.

Pearson’s correlation was used to examine the relations between quantitative variables of the population. The stronger the association of the two variables, the closer the Pearson correlation coefficient, *r*, was to either +1 or −1, depending on whether the relationship was positive or negative, respectively.

Linear regression models, unadjusted and adjusted for confounders (adjusted model 1: age, sex; adjusted model 2: age, sex, WC) were then performed to quantify associations showing significant correlations. The z-BMI, VO_2_max, HGS and RMR were used as the dependent variables. Parameters of metabolic health and eating behavior were used as predictor variables. Regression beta coefficients, *b*, were assessed explaining the degree of change in the outcome variable for every 1-unit of change (increase or decrease depending on whether the relationship is positive or negative) in the predictor variable.

## 4. Results

### 4.1. Children’s General Characteristics

**Figure 1:**
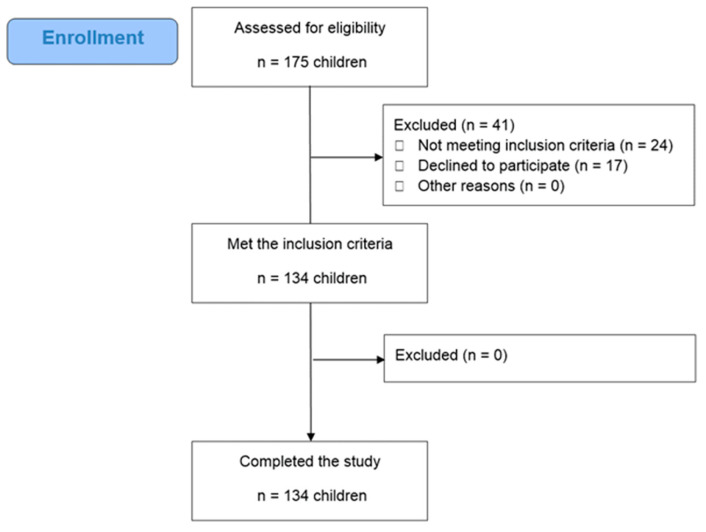
Study flow chart.

As shown in Figure 1, a total of 175 children were assessed to participate in the study. When study criteria were applied, 20 children were excluded due to seasonal influenza and 4 children due to the presence of chronic diseases (3 with diabetes type 1 and one child with celiac disease). Additionally, 17 children withdrew from the study as their parents (or guardians) did not respond to our communication efforts or were willing to discontinue for personal reasons. Consequently, 134 children completed the study. All children were Greek, lived in urban area of Attica and belonged to a middle-class socio-economic status.

Descriptive characteristics regarding anthropometry, CRF, physical strength, biochemical markers and eating habits in the whole sample (n = 134) are shown in Table 1. The mean age of participants was 8.8 years and 57.5% were boys. Based on z-BMI, 81.7% of children had a normal weight, while 35.1% had overweight/obesity. Only two children (1.9%) belonged to the category of underweight (z-BMI < −2). Using the WHtR ≥ 0.5 cut-off [23], abdominal adiposity was found in 36.2% of children with overweight/obesity and 2.4% in normal weight children. Most children consumed breakfast (75.4%) and snacked during school time (73.1%).

Table 1 also presents mean levels for all tested variables between sexes. Concerning anthropometry, boys showed significantly higher z-scores of weight (*p* = 0.042) than girls. Additionally, boys demonstrated higher CRF and physical strength compared to girls, as indicated by VO_2_max (*p* = 0.003) and HGS (*p* = 0.018) respectively. Biochemical markers and eating habits did not differ between sexes.

### 4.2. Differences of Tested Variables According to Body Weight Status

Table 2 presents mean differences for all tested variables between normal weight children and children with overweight/obesity. In regard to anthropometry, children with overweight/obesity demonstrated higher z-BW for age (*p* < 0.001), WC (*p* < 0.001), WHtR (*p* < 0.001) and FM % (*p* < 0.001) compared to their normal weight counterparts. On the contrary, FFM % (*p* < 0.001) and TBW % (*p* < 0.001) were significantly lower in children with overweight/obesity. Additionally, correlation analysis showed that z-BMI was strongly and positively correlated with WC (r = 0.722, *p* < 0.001), WHtR (r = 0.637, *p* < 0.001), FM % (r = 0.725, *p* < 0.001), while a strong and inverse correlation with FFM % (r = −0.728, *p* < 0.001) and TBW % (r = −0.623, *p* < 0.001) was detected. A negative correlation with plasma alpha-tocopherol was also recorded (r = −0.286, *p* < 0.001).

Mean value of RMR (kcal/kg/day) was lower in participants with overweight/obesity compared to those with normal weight (*p* < 0.001). At the same time RMR was positively correlated with FFM % (r = 0.650, *p* < 001) and negatively correlated with FM % (r = −0.642, *p* < 0.001) and z-BMI (r = −0.575, *p* < 0.001).

With respect to CRF, VO_2_max was lower in children with overweight/obesity than those of normal weight (*p* < 0.001). VO_2_max was positively correlated with FFM % (r = 0.326, *p* < 0.001) and negatively correlated with FM % (r = −0.349, *p* < 0.001) and z-BMI (r = −0.195, *p* = 0.029).

HGS did not differ between the normal weight and the overweight/obesity group (*p* = 0.371), but was strongly and positively correlated with FFM (r = 0.869, *p* < 0.001) and RMR (r = 0.567, *p* < 0.001). HGS tended to correlate significantly with HDL-C (r = 0.207, *p* = 0.055) and showed a significantly negative correlation with TG/HDL-C ratio (r = −0.224, *p* = 0.037).

Blood lipid profile and antioxidant vitamin levels did not differ between groups. However, a negative correlation of z-BMI with plasma vitamin E was identified (r = −0.286, *p* = 0.001).

### 4.3. Differences between Tested Variables According to Breakfast Eating Behavior

As regards breakfast, this was consumed exclusively at home. Children who reported not having breakfast at home, all skipped this meal in the morning. According to Table 2, the proportion of children having breakfast regurarly was greater in normal weight children than that of the overweight/obesity group (*p* = 0.009). The proportion of children who snacked during school did not differ between normal weight and overweight/obesity groups.

Lower mean values of z-BMI, WC, WHtR and FM %, and higher FFM % were found in children who had breakfast daily or most of the days, than those who skipped this meal (Table 3). It is noteworthy that RMR was found to be greater in children having breakfast than those who did not (*p* = 0.035). Mean values of HGS and VO_2_max did not differ between these two groups.

### 4.4. Linear Regression Analyis

Table 4 presents outcomes of linear regression models (unadjusted model, adjusted model 1, and adjusted model 2), regarding the associations of z-BMI, RMR, CRF and HGS with eating behavior and parameters of metabolic health. In agreement with correlation analysis, z-BMI was significantly associated with body composition (FM and FFM), even when controlling for age, sex, and WC. An inverse association with antioxidant plasma vitamin E (alpha-tocopherol) was also evident in both adjusted regression models. With regard to eating behavior, z-BMI was negatively associated with having breakfast regularly at home (*p* = 0.016), even when adjusted to age and sex (*p* = 0.010).

Similarly, RMR and HGS were significantly associated with FFM in all tested regression models, while CRF, as indicated by VO_2_max was negatively associated with FM even after controlling for age, sex and WC. RMR was positively associated with eating breakfast on a regular basis (*p* = 0.020), and after adjusting for sex, age and WC, this association persisted (*p* = 0.05). Furthermore, HGS was inversely associated with TG/HDL-C (*p* = 0.037), but this association discontinued in both adjusted regression models.

## 5. Discussion

In the present study, we explored the role of CRF, physical strength and dietary habits in different food environments, either breakfast exclusively at home or snacking exclusively at school, in metabolic health of primary children in Greece. To the best of our knowledge, this is the first study aiming at investigating VO_2_max, HGS and RMR, and how these factors simultaneously correlate to adiposity, antioxidant status, blood lipid profile and eating habits of Greek children. For this purpose, 134 children, aged 6 to 11 years old, visited our clinical site along with their parents to get a full screening regarding anthropometry (i.e., BMI, RMR, body circumferences, bod composition), cardiorespiratory fitness (i.e., VO_2_max), physical strength (i.e., HGS), blood analysis (i.e., blood lipids and antioxidant vitamins A, E, C) and assessment of particular eating behavior.

In the European region childhood obesity is a major health concern. Outcomes of the WHO European Childhood Obesity Surveillance Initiative (COSI) recording obesity trends in children aged 6–9 years during 2015–2017 have revealed that overall, 28.7% of boys and 26.5% of girls had overweight/obesity [35]. In fact, children of Southern Europe were characterized as the heaviest, while the mean z-score BW-for-age of Greek children was among the highest [35]. Additionally, Greece was found to possess the highest z-BMI-for-age for boys [35]. In the same study, sex affected obesity prevalence. Based on the WHO growth reference data, Greek boys had a prevalence of overweight/obesity of 42% [95% Confidence Interval (CI) 38.5–45.5] compared to girls, who had a prevalence of 37.8% [95% CI 34.5–41.1] [35]. Similar to the previous study, we found that almost 35% of participants were classified as overweight or obese, with the z-score BW-for-age being substantially higher in boys than in girls.

With regard to abdominal adiposity prevalence, data from a large cohort study in Greece during 2015 showed a prevalence of about 95% in children with obesity, 66% in children with overweight and 12% in normal weight children [36]. For the prediction of abdominal adiposity, authors used the established cut-off point of WHtR ≥ 0.5 [36]. In the current study, we found that abdominal adiposity was less common, occurring at a rate of 36.2% in children who were overweight or obese and 2.4% in children who were of normal weight. These variations may be attributed to differences in sample sizes, geographic regions, as well as the declining prevalence of childhood obesity since 2010, as many preventive interventions targeting primary schoolchildren in Greece have taken place [37].

According to some research, abdominal adiposity in children and adolescents can predict the likelihood of developing cardiometabolic abnormalities such as dyslipidemia, type 2 diabetes, hypertension, and coronary artery disease more accurately than BMI alone [38]. For instance, abdominal adiposity has been linked to childhood dyslipidaemia, regardless of BMI [39]. Abdominal adiposity in children is determined by several indexes, mainly WC and WHtR [38]. Compared to WC, the WHtR is considered more useful, since the 0.5 cut-off value is universal and would help standardize clinical practice independently of age, sex, height and race [40,41]. Additionally, the role of WHtR for the prediction of cardiometabolic risk factors in children has been well established [41]. Results from an observational study in Italian primary schoolchildren showed that WHtR was positively correlated with BMI [42]. In line with this, our results indicated that z-BMI was strongly and positively correlated with WC and WHtR. This observation was confirmed by body composition analysis, as children with overweight/obesity demonstrated higher mean values of FM % and lower values of FFM % compared to their normal weight counterparts. In fact, z-BMI was strongly and positively correlated with FM % and negatively correlated with FFM % and TBW %, highlighting the vital role of assessing body composition in children [43,44].

Additionally, we recorded an inverse correlation of z-BMI with plasma vitamin E, probably indicating the negative impact of overweight/obesity on redox balance and metabolic health [45]. To assess vitamin E, we quantified plasma alpha-tocopherol, the main form of vitamin E in the human body and the most metabolically active [46]. Vitamin E is mostly accumulated in the adipose tissue (~90%), as well as in the liver and the adrenal glands [46]. There is evidence that vitamin E supplementation significantly increases alpha-tocopherol in plasma, reaching a maximum concentration at 8 h post-ingestion followed by substantial decline [47]. This observation could explain the non-detectable differences for vitamin E between the normal weight and overweight/obesity group in our study. Metabolic abnormalities, however, seem to affect vitamin’s bioavailability. According to a randomized controlled study, alpha-tocopherol concentration in plasma was lower in adults with metabolic syndrome compared to healthy individuals. It is suggested that the presence of systemic inflammation and oxidative stress affects intestinal absorption of the vitamin and impairs hepatic alpha-tocopherol trafficking [48]. The role of vitamin E in childhood obesity has been investigated in randomized clinical trials. For instance, it has been shown that oral vitamin E administration in children with obesity-related liver disease normalized or stabilized serum aminotransferase and alkaline phosphatase levels [49,50]. Therefore, the observed negative association between z-BMI and vitamin E in our study, even after controlling for age, sex and adiposity (WC), could imply the pivotal role of vitamin E dietary intake in child overweight/obesity.

It is well documented that absolute RMR (kJ/day) is higher in children with obesity than in lean ones [51,52,53], an outcome also evident in the present study. However, RMR is directly related to body weight, which in turn accounts for about 70 to 80% of the variance in RMR [51]. To this end, we also presented RMR output as kcal/kg of BW/day, and results showed that the mean value was greater in normal weight participants compared to those with overweight/obesity, verifying the indisputable role of body size in child’s resting energy metabolism. Furthermore, body composition is considered a main determinant of RMR. Zwiauer et al. showed a strong correlation of RMR with FFM in children [52], which was also the case in the present study. We also recorded a negative correlation of RMR with FM, while absolute RMR (kcal/day) was greater in boys than in girls, an observation conforming that FM and sex are additional determinants of RMR [53].

Nevertheless, little is known regarding the association of overweight/obesity with CRF, namely VO_2_max. In 2021, Grzyb et al. have shown that children and adolescents with obesity demonstrated similar VO_2_max values compared to those with overweight, but significantly greater than children of normal weight [54]. There is evidence that augmentation of VO_2_max in adults is related to lower FM and WC [8,9,10], but in the case of children, data are still limited. Morinder et al. have demonstrated that VO_2_max was a stronger marker for insulin sensitivity compared to FM in severely obese children and adolescents, an outcome that may implies the imperative role of CRF measurement for predicting metabolic syndrome in children [55]. Likewise, in the present study the mean VO_2_max was higher in the group with overweight/obesity than in lean children, also showing a negative association with FM, even after adjusting for age, sex and WC.

VO_2_max is directly correlated with HGS, a strong predictor of cardiovascular health in both young adults and children [13,14,16]. Recently, children with overweight/obesity showed higher HGS and lower CRF compared to their normal weight counterparts [16,56]. In the present study, HGS did not differ between the two groups, but was strongly and positively correlated with % FFM and RMR, verifying that HGS can be used as a metabolic health indicator in children [57]. We also reported that HGS was negatively associated with TG/HDL-C ratio, a well-known marker of cardiometabolic health [58]. When adjusted to adiposity (WC), HGS showed no association with TG/HDL-C. This is also confirmed by previous studies, as the negative impact of overweight and obesity on cardiometabolic health in both children and adults is well established [56].

It has been suggested that certain meals are important for children’s weight status. Breakfast consumption has been linked to a healthier BMI, according to observational studies in the Greek child population [59,60]. Similarly, the present study indicated that children who ate breakfast had lower z-BMI, WHtR and FM than those who did not. Moreover, RMR was positively associated with having breakfast adjusting for age, sex and adiposity, an observation that has been reported by Tambalis for Greek children [61]. Breakfast intake in the current survey was restricted to eating at home. There is evidence that increasing breakfast consumption at home may limit the amount of unhealthful snack foods children consume later in the day, especially for those children on greater socioeconomic disadvantage [62]. Breakfast at home environment is considered more nutritious and access to nutritious meals is a fundamental determinant of health. To this end, it is suggested that parents should be advised to provide their children a healthful breakfast at home in order to attain a healthy body weight [62].

The present study also had some limitations. We are aware that the sample size is relatively small. To overcome this, we applied strict inclusion and exclusion criteria, while all appointed researchers were well trained to avoid measurement errors. Additionally, measurements and interview sessions for each child were conducted by the same researchers to omit bias. Furthermore, we did not evaluate levels of physical activity. In addition, we did not assess nutrient composition of breakfast meals. In the future, large case-control studies are needed to investigate the implication of nutrients and non-nutrients obtained from breakfast in children’s CRF and metabolic health.

## 6. Conclusions

In the present study, we explored the role of cardiorespiratory fitness, physical strength and particular meal consumption in metabolic health of 134 primary children in Greece. Results indicated that z-BMI had a positive correlation with WC, WHtR, and fat mass but a negative correlation with CRF, antioxidant status (as measured by blood vitamin E levels), and frequent breakfast consumption. According to the TG/HDL-C ratio, HGS had a negative correlation with dyslipidemia but a positive correlation with FFM. A positive association of RMR with eating breakfast at home on a regular basis was also found, even after adjusting of sex, age and adiposity. Therefore, CRF, physical strength and breakfast intake before school are promising key modulators of cardiometabolic health in Greek children.

## Figures and Tables

**Table 1:** Children characteristics in the whole sample and on different sexes.

Parameters	Enrolled (N = 134)	Boys (N = 77)	Girls (N = 57)	*p*-Value
**Anthropometry**				
Age (years); mean (SD)	8.8 (1.8)	8.8 (1.7)	8.9 (1.8)	0.709
Height (m); mean (SD)	1.4 (0.1)	1.4 (0.1)	1.4 (0.1)	0.577
Weight (kg); mean (SD)	33.6 (10.0)	34.4 (10.9)	32.6 (8.7)	0.326
BMI (kg/m^2^); mean (SD)	17.8 (3.1)	18.1 (3.4)	17.5 (2.5)	0.301
Z-score weight; mean (SD)	0.9 (1.2)	1.0 (1.2)	0.6 (1.0)	**0.042**
Z-score height; mean (SD)	0.8 (1.1)	0.9 (1.1)	0.6 (1.0)	0.064
Z-score BMI; mean (SD)	0.6 (1.2)	0.7 (1.3)	0.4 (1.1)	0.156
Weight status based on z-score BMI; N (%)				
<−2, underweight	2 (1.9)	1 (1.3)	1 (1.8)	**-**
−2 to +0.99, normal weight	85 (81.7)	48 (62.3)	37 (64.9)	0.531
≥1, overweight/obese	47 (35.1)	28 (36.4)	19 (33.3)	0.393
WC (cm); mean (SD)	61.1 (8.7)	62.0 (9.5)	59.8 (7.4)	0.149
WHtR; mean (SD)	0.4 (0.6)	0.4 (0.7)	0.4 (0.0)	0.564
FM (%); mean (SD)	23.7 (9.5)	22.4 (10.4)	25.4 (7.9)	0.070
FM (kg); mean (SD)	8.6 (5.8)	8.5 (6.7)	8.8 (4.3)	0.777
FFM (%); mean (SD)	76.3 (9.6)	77.5 (10.6)	74.6 (7.9)	0.082
FFM (kg); mean (SD)	25.0 (5.7)	25.8 (5.5)	24.0 (5.8)	0.064
TBW (%); mean (SD)	63.7 (9.2)	64.6 (9.9)	62.4 (8.0)	0.169
**Cardiorespiratory fitness and strength**				
RMR (kcal/day); mean (SD)	1565.5 (278.9)	1628.0 (276.2)	1479.8 (261.4)	**0.003**
RMR (kcal/kg/day); mean (SD)	49.0 (10.0)	49.6 (9.8)	48.1 (10.3)	**0.414**
VO_2_max (mL/kg/min); mean (SD)	49.9 (9.5)	52.5 (10.2)	47.0 (7.6)	**0.003**
HRmax (bpm); mean (SD)	202.9 (11.3)	201.9 (10.5)	204.2 (12.3)	0.297
HGS (kg); mean (SD)	15.2 (5.2)	16.3 (5.4)	13.2 (4.7)	**0.018**
**Blood indices**				
Vit A (mg/L); mean (SD)	0.3 (0.2)	0.3 (0.1)	0.3 (0.2)	0.852
Vit E (mg/L); mean (SD)	10.9 (2.6)	10.9 (2.8)	10.8 (2.4)	0.859
Vit C (mg/L); mean (SD)	5.6 (1.3)	5.6 (1.0)	5.6 (1.7)	0.826
Total cholesterol (mg/dL); mean (SD)	164.3 (26.8)	166.1 (25.1)	161.8 (29.1)	0.366
HDL-C (mg/dL); mean (SD)	53.2 (12.2)	53.5 (11.1)	52.7 (12.6)	0.729
LDL-C (mg/dL); mean (SD)	99.6 (23.6)	101.5 (22.4)	97.1 (26.2)	0.288
TG (mg/dL); mean (SD)	57.4 (19.1)	55.5 (16.5)	59.9 (22.0)	0.196
TG/HDL-C ratio; mean (SD)	1.2 (0.7)	1.1 (0.5)	1.3 (0.8)	0.268
**Meals**				
Daily breakfast (YES/NO); N (%)	101 (75.4)/23 (17.2)	58 (75.3)/13 (16.9)	43 (75.4)/10 (17.5)	0.937
Daily snacking at school (YES/NO); N (%)	98 (73.1)/22 (16.4)	52 (67.5)/15 (19.5)	46 (80.7)/7 (12.3)	0.197

Data are presented as counts (%) or means (standard deviation) (SD). For continuous values, the independent-samples *t*-test was conducted to compare means between sexes. For categorical variables, the chi-square test was used. Level of statistical significance was set at *p* ≤ 0.05. Bold *p*-values represent statistically significant differences between groups. BMI, body mass index; WC, waist circumference; WHtR, waist to height ratio; FM, fat mass; FFM, fat-free mass; TBW, total body water; RMR, resting metabolic rate; HRmax, maximal heart rate; HGS, handgrip strength; HDL-C, high-density lipoprotein-cholesterol; LDL-C, low-density lipoprotein-cholesterol; TG, triglycerides.

**Table 2:** Differences in tested variables based on body weight status.

Parameters: Mean (SD)	Normal Weight (N = 85)	Overweight/Obese (N = 47)	*p*-Value
**Anthropometry**			
z-score BMI	−0.05 (0.7)	1.9 (0.8)	**<0.001**
z-score height	0.6 (1.0)	1.1 (1.1)	**0.002**
z-score weight	0.3 (0.8)	2.0 (0.9)	**<0.001**
Age (years)	8.8 (1.8)	8.8 (1.7)	0.897
Height (m)	1.3 (0.1)	1.4 (0.1)	0.076
Weight (kg)	29.9 (7.3)	40.7 (10.8)	**<0.001**
BMI (kg/m^2^)	16.2 (1.5)	20.9 (2.8)	**<0.001**
WC (cm)	57.4 (5.8)	68.1 (8.9)	**<0.001**
WHtR	0.4 (0.1)	0.5 (0.05)	**<0.001**
FM (%)	18.9 (6.4)	32.4 (8.2)	**<0.001**
FM (kg)	5.6 (2.7)	13.7 (6.5)	**<0.001**
FFM (%)	81.1 (6.4)	67.5 (8.3)	**<0.001**
FFM (kg)	24.0 (5.6)	27.0 (5.5)	**0.004**
TBW (%)	67.5 (7.7)	56.4 (7.0)	**<0.001**
**Cardiorespiratory fitness and strength**			
RMR (kcal/kg/day)	52.9 (9.0)	42.3 (8.1)	**<0.001**
VO_2_max (mL/kg/min)	52.4 (8.4)	46.1 (10.1)	**<0.001**
HRmax (bpm)	202.8 (11.7)	203.0 (10.7)	0.932
HGS (kg)	14.8 (4.8)	15.8 (5.9)	0.371
**Blood indices**			
Vit A (mg/L)	0.3 (0.1)	0.3 (0.2)	0.582
Vit E (mg/L)	10.9 (2.4)	10.5 (2.9)	0.435
Vit C (mg/L)	5.5 (1.1)	5.7 (1.7)	0.591
Total cholesterol (mg/dL)	165.0 (27.5)	163.3 (26.0)	0.736
HDL-C (mg/dL)	53.2 (11.9)	53.3 (12.8)	0.968
LDL-C (mg/dL)	100.2 (24.5)	98.6 (22.4)	0.719
TG (mg/dL)	57.9 (20.0)	56.9 (17.5)	0.764
TG/HDL-C ratio	1.2 (0.7)	1.2 (0.6)	0.859
**Meals**			
Daily breakfast (YES/NO); N (%)	68 (80.0)/9 (10.6)	31 (66.0)/14 (29.8)	**0.009**
Daily snacking at school (YES/NO); N (%)	62 (72.9)/15 (17.6)	34 (72.3)/7 (14.9)	0.478

Data are presented as means (standard deviation) (SD) or counts (%). The independent-samples *t*-test was conducted to compare variable means between children of overweight/obesity and normal weight. To compare eating behavior frequencies between groups, the chi-square test was used. Level of statistical significance was set at *p* ≤ 0.05. Bold *p*-values represent statistically significant differences between groups. BMI, body mass index; WC, waist circumference; WHtR, waist to height ratio; FM, fat mass; FFM, fat-free mass; TBW, total body water; RMR, resting metabolic rate; HRmax, maximal heart rate; HGS, handgrip strength; HDL-C, high-density lipoprotein-cholesterol; LDL-C, low-density lipoprotein-cholesterol; TG, triglycerides.

**Table 3:** Differences in tested variables based on eating behavior.

Parameters: Mean Values (SD)	Consuming Breakfast		
	Daily or Most of the Days (N = 101)	Sometimes/No (N = 23)	*p*-Value
z-score BMI	0.5 (1.3)	1.2 (1.2)	**0.012**
z-score height	0.8 (1.1)	0.6 (1.2)	0.393
z-score weight	0.8 (1.2)	1.2 (1.3)	0.175
Age (years)	8.6 (1.8)	9.4 (1.3)	**0.042**
Height (m)	1.3 (0.1)	1.4 (0.1)	0.165
Weight (kg)	32.3 (9.4)	37.9 (10.0)	**0.012**
BMI (kg/m^2^)	17.4 (3.0)	19.5 (3.3)	**0.002**
WC (cm)	60.0 (8.3)	65.5 (9.6)	**0.006**
WHtR	0.4 (0.1)	0.5 (0.1)	**0.025**
FM (%)	23.1 (9.5)	27.7 (9.2)	**0.041**
FM (kg)	8.2 (5.5)	11.1 (6.5)	**0.029**
FFM (%)	76.9 (9.5)	72.4 (9.2)	**0.044**
FFM (kg)	24.2 (5.3)	26.8 (5.0)	**0.035**
TBW (%)	64.0 (9.4)	60.2 (7.9)	0.082
RMR (kcal/kg/day)	49.6 (9.9)	44.6 (7.3)	**0.035**
VO_2_ max (mL/kg/min)	49.7 (10.1)	48.6 (6.3)	0.642
HRmax (bpm)	202.2 (12.0)	205.2 (8.5)	0.304
HGS (kg)	14.5 (5.0)	16.2 (3.8)	0.159
Vit A (mg/L)	0.3 (0.2)	0.3 (0.1)	0.531
Vit E (mg/L)	10.9 (2.8)	10.7 (2.3)	0.769
Vit C (mg/L)	5.5 (1.4)	5.7 (1.1)	0.733
Total cholesterol (mg/dL)	163.8 (27.7)	163.0 (23.5)	0.895
HDL (mg/dL)	52.8 (12.1)	54.9 (11.8)	0.460
LDL (mg/dL)	99.5 (24.1)	96.9 (22.1)	0.629
TG (mg/dL)	57.2 (19.8)	56.1 (15.6)	0.814
TG/HDL-C ratio	1.2 (0.7)	1.1 (0.4)	0.524

Data are presented as counts (%) or means (standard deviation) (SD). Independent-samples *t*-test was conducted to compare variable means between groups. Level of statistical significance was set at *p* ≤ 0.05. Bold *p*-values represent statistically significant differences between groups. BMI, body mass index; WC, waist circumference; WHtR, waist to height ratio; FM, fat mass; FFM, fat-free mass; TBW, total body water; RMR, resting metabolic rate; HRmax, maximal heart rate; HGS, handgrip strength; HDL-C, high-density lipoprotein-cholesterol; LDL-C, low-density lipoprotein-cholesterol; TG, triglycerides.

**Table 4:** Regression analysis addressing the associations between metabolic health factors and eating behavior.

Tested Associations	Unadjusted Model		Adjusted Model 1		Adjusted Model 2	
	Beta	*p*-Value	Beta	*p*-Value	Beta	*p*-Value
z-BMI						
FFM (kg)	0.414	**<0.001**	0.823	**<0.001**	0.258	**0.009**
FM (kg)	0.709	**<0.001**	0.845	**<0.001**	0.461	**<0.001**
Plasma vitamin E (mg/L)	−0.286	**0.001**	−0.293	**0.001**	−0.161	**0.004**
Eating breakfast regularly	−0.218	**0.016**	−0.237	**0.010**	−0.082	0.173
VO2max (mL/kg/min)						
FFM (kg)	0.135	0.143	0.122	0.367	0.092	0.560
FM (kg)	−0.258	**0.005**	−0.425	**<0.001**	−0.602	**<0.001**
HGS (kg)						
FFM (kg)	0.869	**<0.001**	0.658	**<0.001**	0.627	**<0.001**
FM (kg)	0.450	**<0.001**	0.248	**<0.001**	0.066	0.520
HDL-C (mg/dL)	0.207	0.055	0.118	0.094	0.063	0.343
TG/HDL-C	−0.224	**0.037**	−0.103	0.142	−0.075	0.247
RMR (kcal/day)						
FFM (kg)	0.704	**<0.001**	0.638	**<0.001**	0.464	**<0.001**
FM (kg)	0.504	**<0.001**	0.335	**<0.001**	0.028	0.812
Eating breakfast regularly	0.212	**0.020**	0.213	**0.005**	0.196	**0.050**

Level of statistical significance was set at *p* ≤ 0.05. Bold *p*-values represent statistically significant associations between variables. Model 1: adjusted for age and sex. Model 2: adjusted for age, sex and WC. BMI, body mass index; WC, waist circumference; FM, fat mass; FFM, fat-free mass; RMR, resting metabolic rate; HGS, handgrip strength; HDL-C, high-density lipoprotein-cholesterol; TG, triglycerides.

## Data Availability

Data is unavailable due to privacy or ethical restrictions.
